# Serum Protein Biomarker Findings Reflective of Oxidative Stress and Vascular Abnormalities in Male, but Not Female, Collision Sport Athletes

**DOI:** 10.3389/fneur.2020.549624

**Published:** 2020-09-30

**Authors:** Brendan P. Major, Stuart J. McDonald, William T. O'Brien, Georgia F. Symons, Meaghan Clough, Daniel Costello, Mujun Sun, Rhys D. Brady, Jesse Mccullough, Roxanne Aniceto, I-Hsuan Lin, Meng Law, Richelle Mychasiuk, Terence J. O'Brien, Denes V. Agoston, Sandy R. Shultz

**Affiliations:** ^1^Department of Neuroscience, Monash University, Melbourne, VIC, Australia; ^2^Department of Physiology, Anatomy, and Microbiology, La Trobe University, Melbourne, VIC, Australia; ^3^Department of Medicine, The University of Melbourne, Parkville, VIC, Australia; ^4^Department of Anatomy, Physiology, and Genetics, Uniformed Services University, Bethesda, MD, United States; ^5^Department of Electrical and Computer Systems Engineering, Monash University, Melbourne, VIC, Australia; ^6^Departments of Neurological Surgery and Biomedical Engineering, University of Southern California, Los Angeles, CA, United States

**Keywords:** mild TBI, concussion, sub-concussion, vascular injury, cerebrovascular, VEGF–vascular endothelial growth factor, 4-HNE

## Abstract

Studies have indicated that concussive and sub-concussive brain injuries that are frequent during collision sports may lead to long-term neurological abnormalities, however there is a knowledge gap on how biological sex modifies outcomes. Blood-based biomarkers can help to identify the molecular pathology induced by brain injuries and to better understand how biological sex affects the molecular changes. We therefore analyzed serum protein biomarkers in male (*n* = 50) and female (*n* = 33) amateur Australian rules footballers (i.e., Australia's most participated collision sport), both with a history of concussion (HoC) and without a history of concussion (NoHoC). These profiles were compared to those of age-matched control male (*n* = 24) and female (*n* = 20) athletes with no history of neurotrauma or participation in collision sports. Serum levels of protein markers indicative of neuronal, axonal and glial injury (UCH-L1, NfL, tau, p-tau, GFAP, BLBP, PEA15), metabolic (4-HNE) and vascular changes (VEGF-A, vWF, CLDN5), and inflammation (HMGB1) were assessed using reverse phase protein microarrays. Male, but not female, footballers had increased serum levels of VEGF-A compared to controls regardless of concussion history. In addition, only male footballers who had HoC had increased serum levels of 4-HNE. These findings being restricted to males may be related to shorter collision sport career lengths for females compared to males. In summary, these findings show that male Australian rules footballers have elevated levels of serum biomarkers indicative of vascular abnormalities (VEGF-A) and oxidative stress (4-HNE) in comparison to non-collision control athletes. While future studies are required to determine how these findings relate to neurological function, serum levels of VEGF-A and 4-HNE may be useful to monitor subclinical neurological injury in males participating in collision sports.

## Introduction

There is growing evidence that exposure to repetitive head impacts (RHI) during participation in collision sports are associated with short- and long-term neurological abnormalities (1–3). Although much research has focused on concussion, a form of mild traumatic brain injury (mTBI) that typically results in the rapid onset of short-lived impairment in neurological function (4), there is growing evidence that sub-concussive impacts that do not result in overt neurological impairment may also contribute to chronic consequences, particularly if experienced repeatedly (5). Nonetheless, the potential effects of RHI exposure remain poorly understood. Moreover, there is a lack of knowledge related to how males and females may differ in their respond to RHIs (6), as well as the pathobiological mechanisms that contribute to functional consequences (7, 8). Blood-based protein biomarkers can help to identify the molecular changes resulting from concussive and sub-concussive brain impacts, and how biological sex influence the molecular responses (3, 9, 10).

There are a number of pathophysiological processes that have been implicated in RHIs, including oxidative stress, inflammation, and injury to neurons, axons, glia, and cerebrovasculature (11). Serum levels of protein biomarkers have the potential to improve the understanding of these processes in RHI (9, 10, 12–15). Although the majority of blood biomarker studies have focused on the acute aftermath of concussion, there is initial evidence that there are chronic systemic changes in athletes with a history of RHI exposure. For example, a preliminary study found elevated plasma levels of inflammatory markers in healthy university athletes with a history of multiple concussions (16). Notably, none of the athletes in this study had reported a concussion within a year prior to the testing and the affected markers were different between male and female athletes. These early findings suggest that RHI exposure in collision sports may trigger a lasting inflammatory response, and that the response is affected by the biological sex.

To provide additional insight into the pathophysiological consequences of RHI exposure, and examine how biological sex may modify this response, this study examined the effect of playing collision sports on the serum levels of protein biomarkers indicative of neuronal, axonal, glial and vascular injuries, inflammation, and oxidative stress in male and female amateur Australian rules footballers both with a history of concussion (HoC) and without a history of concussion (NoHoC).

## Methods

### Participants

A total of 83 (male = 50, female = 33) amateur Australian rules football players were recruited during pre-season from clubs competing in the Victorian Amateur Football Association from 2017 to 2019. Players participating in multiple seasons were only included once (i.e., the first season of participation). Men's and women's leagues follow similar full collision rules (3), which provides the opportunity to study sex differences within the same sport. For a more detailed description of Australian football rules and gameplay please see previous publication (3). Forty-four (male = 24 and female = 20) sex, age, and education matched control athletes with no history of brain trauma or engagement in collision sports, were also recruited from local amateur tennis, cricket, track, and field hockey clubs. Individuals with a history of neurosurgery or severe psychiatric disturbances were excluded. To minimize any confounding effects of recent brain injuries, Australian rules football players who had sustained a concussion in the past 6 months were excluded, and all testing was performed during the off season. The Melbourne Health Human Ethics committee approved study procedures, and all participants provided written informed consent.

### Demographic and Concussion History

The sports concussion assessment tool (SCAT) and an additional questionnaire were administered by a trained research assistant to each participant pertaining to demographics, history of concussion, sporting history, education history, as well as any learning difficulties.

### Serum Protein Markers

Ten mL of whole blood was collected using standard phlebotomy procedures into a BD Vacutainer® SST™ II Advance tube for serum preparation. The tube was inverted several times and allowed to clot at room temperature for 30 min prior to centrifugation at 1,500 g for 10 min to separate serum. Serum was then transferred into 0.5 mL aliquots, flash-frozen and stored at −80°C.

Serum protein levels of ubiquitin carboxyl-terminal hydrolase L1 (UCH-L1), neurofilament light chain (NfL), phosphorylated tau (pTau), tau, glial fibrillary acidic protein (GFAP), brain lipid-binding protein (BLBP), astrocytic phosphoprotein PEA15 (PEA15), 4-hydroxynonenal (4-HNE), high mobility group box protein 1 (HMGB1), vascular endothelial growth factor-A (VEGF-A), Claudin 5 (CLDN5), and von Willebrand factor (vWF), were analyzed with reverse phase protein microarrays (RPPM, Table 1).

**Table 1:** List of serum proteins, associated pathophysiology, and antibody details for RPPM analysis.

**Marker**	**Associated pathophysiology**	**Primary Ab (company, product #, dilution)**	**Secondary Ab (company, product #, dilution)**
UCH-L1	Elevated levels indicate neuronal damage	Cell Signaling, 11896, 1:100	Thermo Fisher, A27041, 1:10,000 (Gt anti-Rb)
NfL	Elevated levels indicate axonal damage	ProteinTech, 12998-1-AP, 1:100	Thermo Fisher, A27041, 1:10,000 (Gt anti-Rb)
pTau	Elevated levels indicate axonal pathology and neurodegeneration	Sigma Aldrich, SAB4504563, 1:100	Thermo Fisher, A27041, 1:10,000 (Gt anti-Rb)
Tau	Elevated levels indicate axonal damage	Cell Signaling, 4019, 1:100	Thermo Fisher, A28182, 1:10,000 (Gt anti-Ms)
GFAP	Elevated levels indicate astroglia damage	Abcam, ab7260, 1:1000	Thermo Fisher, A27041, 1:10,000 (Gt anti-Rb)
BLBP	Elevated levels indicate astroglia damage	EMD Millipore, ABN14, 1:100	Thermo Fisher, A27041, 1:10,000 (Gt anti-Rb)
PEA15	Elevated levels indicate astroglia damage	Cell Signaling, 2780, 1:100	Thermo Fisher, A27041, 1:10,000 (Gt anti-Rb)
4-HNE	Elevated levels indicate oxidative stress	EMD Millipore, 393207, 1:100	Thermo Fisher, A27041, 1:10,000 (Gt anti-Rb)
HMGB1	Elevated levels indicate inflammation	Abcam, ab79823, 1:100	Thermo Fisher, A27041, 1:10,000 (Gt anti-Rb)
VEGF-A	Elevated levels indicate vascular injury	Abcam, ab53465, 1:100	Thermo Fisher, A27041, 1:10,000 (Gt anti-Rb)
CLDN5	Elevated levels indicate vascular injury	EMD Millipore, ABT45, 1:200	Thermo Fisher, A27041, 1:10,000 (Gt anti-Rb)
vWF	Elevated levels indicate vascular/endothelial injury	Abcam, ab181871, 1:100	Thermo Fisher, A27041, 1:10,000 (Gt anti-Rb)

*UCH-L1, ubiquitin carboxyl-terminal hydrolase L1; NfL, neurofilament light chain; pTau, phosphorylated tau; GFAP, glial fibrillary acidic protein; BLBP, brain lipid-binding protein; PEA15, astrocytic phosphoprotein PEA15; 4-HNE, 4-hydroxynonenal; HMGB1, high mobility group box protein 1; VEGF-A, vascular endothelial growth factor A; CLDN5, claudin 5; and vWF, von Willebrand factor*.

Sample preparation, printing, scanning, and data analysis for RPPM were performed as described previously (14, 15). Serum samples for the male analysis (i.e., the first set of samples analyzed) were thawed on ice and 50 μL of 8 × SDS Sample Buffer [35% glycerol,.8% SDS, 1 × TBS, 1 × TCEP Bond Breaker (Reducing agent) (Thermo Scientific, Prod # 1861282); 1 × HALT Protease and Phosphatase Inhibitor (Thermo Scientific, Prod # 77720)], 50 μL of PBS, and 75 μL of T-per Tissue Protein Extraction Reagent (Thermo Fisher, Prod # 78510) were added to 25 μL of sample resulting in a 200 μL sample solution. Serum samples for the female analysis (i.e., the second set of samples analyzed) were thawed on ice and 50 μL of 8× SDS Sample Buffer [35% glycerol, 1.6% SDS, 1× TBS, 1× TCEP Bond Breaker (Reducing agent) (Thermo Scientific, Prod # 1861282); 1× HALT Protease and Phosphatase Inhibitor (Thermo Scientific, Prod # 77720)], 50 μL of PBS, and 75 μL of T-per Tissue Protein Extraction Reagent (Thermo Fisher, Prod # 78510) were added to 25 μL of sample resulting in a 200 μL sample solution. The samples were then denatured at 70°C for 10 min, immediately followed by quenching on ice. Excluding the first and seventh columns, 100 μL of dilution buffer (1 part 8x SDS Sample Buffer; 3 parts T-per Tissue Protein Extraction Reagent) was loaded into each well in a 96-well plate. The 200 μL of denatured samples were pipetted into the first and seventh columns to be horizontally diluted in the 96-well plates, and using a multichannel pipette set to 100 μL, the samples were serially diluted in a 1:2 manner (5-step), yielding the 6 total sample concentrations (the original denatured sample and its five 1:2 serial dilutions). The 96-well round-bottom plates containing the serially-diluted samples were then loaded into the JANUS, along with four empty 384-well plates and RoboRack 200 μL Clear Non-Conductive Tips (PerkinElmer, Prod # 6000681). The JANUS then transferred the serially diluted samples from the 96-well round bottom plates to the 384-well source plates in a predetermined layout. The source plates were subsequently transferred into an Aushon 2470 Arrayer (Aushon Biosystems, Billerica, MA) where serum samples were printed onto ONCYTE AVID nitrocellulose film slides (Grace Bio-Labs, Bend, OR, Prod # 305177). The Aushon 2470 Arrayer was set with 16 pins and programmed for 1 deposition per spot serum; spot diameter was set to 250 nm with spacing between dots at 500 nm on the x-axis and 370 nm on the y-axis, and wash time was set to 2 seconds without delays and humidity set to 80%. The printed slides were placed on an orbital shaker and air-dried for 1 h. After drying, each slide was placed into its own well in a Nunc 4-well dish and was washed three times with 5 mL 1× TBS per slide for 5 min each and blocked with Azure Protein-Free Blocking Buffer (VWR, Prod # 10147-336) for 1 h. The slides were then washed three times with 5 mL 1× TBST per slide and once more with 1x TBS, for 5 min each.

Primary antibodies were validated in conventional Western Blotting technique on binding specificity and diluted with 1x Azure Protein-free buffer in 1.5 mL Eppendorf Tubes to the desired concentration making a final volume of 250 μL (see Table 1 for antibody product and dilution details). Slides were placed atop a paper towel, had their nitrocellulose coating outlined with a hydrophobic pen, and placed into their corresponding labeled wells within a humidity chamber prepared with a paper-towel strip soaked with 1 mL 0.9% saline solution in each well. Then 200 μL of the primary antibody solution was pipetted on to each slide, covered with an mSeries Lifterslip coverslip (white edges down) (Thermo Fisher, Cat #25X60I-M-5439-001-LS) and incubated overnight (8–12 h) at 4°C. The following day, each slide was placed into their own well in a fresh Nunc dish and washed three times with 5 mL 1× TBST followed by a single wash with 5 mL TBS for 5 min each. Secondary antibodies were diluted in 1x Azure Protein-free buffer (1:10,000 dilution), and slides were incubated with 5 mL of their respective secondary antibody solutions in each well of the Nunc dish at room temperature for 1 h. Slides were then washed three times with 5 mL 1× TBST followed by three washes with 5 mL 1× TBS for 5 min each. Slides were dried by placing them nitrocellulose-side up onto a paper towel atop an orbital shaker for 30 min and then loaded into the tray of the Innopsys InnoScan 710-IR scanner for XDR (extended dynamic range) signal acquisition at 785 nm.

Scanner fluorescence data were imported into a Microsoft Excel-based bioinformatics program. After correcting for local background noise, points indiscernible from background were excluded (SNR <2, Net Fluorescence <5) and secondary-only signals were subtracted from corresponding slides. Net intensity vs. dilution was plotted on a log2-log2 scale; each local block of samples was fit individually, using inter-quartile range to exclude outliers outside upper and lower bounds. The slope of the linear portion of the logistic curve was calculated and the line extrapolated back to zero (i.e., the y-intercept), assessing the amount of protein expressed.

### Statistical Analysis

The demographic measures of age, years of education, age commence sport, and years of sport were analyzed with two-way analysis of variance (ANOVA) with sex (male, female) and athlete group (non-collision control, Australian rules footballer with a HoC, Australian rules footballer with NoHoC) as between subject factors, and Tukey's multiple comparisons *post hoc* tests were conducted where appropriate. The measures of age commence collision sport and years of collision sport participation were analyzed with two-way ANOVA with sex (male, female) and concussion history (Australian rules footballers with a HoC, Australian rules footballers with NoHoC) as between subject factors. Differences between males and females with a HoC on the measures of number of previous concussions and time since last concussion were analyzed with Mann–Whitney tests. For RPPM biomarker measures, no between sex comparisons were performed because the male and female serum samples were analyzed on two separate RPPM runs. Therefore, athlete group was used as the between-subjects factor. Normally distributed data was analyzed with a one-way ANOVA. Data that were not normally distributed were analyzed with Kruskal–Wallis tests, and followed by Dunn's multiple comparison *post-hoc* tests when appropriate. Effect sizes were calculated from Kruskal–Wallis tests. Spearman correlation coefficients were calculated between all serum biomarkers. Spearman rank correlation coefficients were also completed to explore the relationships between serum markers, age commence collision sport, and years of exposure to collision sport. Statistical analyses were performed using SPSS (IBM Corp., Armonk, NY, USA) and GraphPad Prism (GraphPad Software, Version 8.10, Inc. La Jolla, CA, USA), with statistical significance defined as *p* < 0.05. Sample size calculations were based on our previous studies that have applied serum protein measures in the context of mTBI (14, 15).

## Results

### Demographics

Demographical, sporting history, and concussion history findings for non-collision controls, Australian rules footballers with NoHoC, and Australian rules footballers with a HoC are presented in Table 2. A main effect of athletic group was found for age (*F*_2, 117_ = 3.94, *p* = 0.02), however no *post-hoc* differences were found. There was a main effect of sex on years of education (*F*_1, 114_ = 4.30, *p* = 0.04; Males > Females). There was a main effect of sex on age commence all sport (*F*_1, 107_ = 4.30, *p* = 0.04; Males > Females). A main effect of sex (*F*_1, 106_ = 5.83, *p* = 0.01; Females > Males), and a sex ^*^ athletic group interaction (*F*_2, 106_ = 3.87, *p* = 0.02) was found for years of sport participation, with HoC males being significantly less than NoHoC females (*p* = 0.005). For age of commencement of collision sport, there was a main effect of sex (*F*_1, 69_ = 44.80, *p* = 0.0001; Males < Females). For years of collision sport participation, there was a main effect of sex (*F*_1, 69_ = 25.76, *p* = 0.0001; Males > Females) and HoC (*F*_1, 69_ = 4.16, *p* = 0.045; HoC > NoHoC). There were no differences between HoC males and HoC females on the measures of previous concussions and time since most recent concussion.

**Table 2:** Demographic results for non-collision sport controls, and Australian rules footballers with and without a history of concussion.

	**Non collision sport controls**		**Australian rules footballers**			
			**No history of concussion**		**History of concussion**	
	**Male**	**Females**	**Male**	**Female**	**Male**	**Female**
*N*	23	19	19	23	31	8
Age	21.8 ± 0.9	22.4 ± 0.7	22.4 ± 0.5	23.5 ± 0.9	23.7 ± 0.5	25.9 ± 0.5
Years education^*^	16.6 ± 0.3	15.9 ± 0.4	16.0 ± 0.3	15.5 ± 0.4	16.9 ± 0.3	16.2 ± 0.7
Age commence all sport^*^	7 ± 0.6	7.2 ± 0.6	8.3 ± 1	5.3 ± 0.8	6.8 ± 0.8	5.3 ± 0.8
Years of all sport^*^	15.0 ± 0.5	13.7 ± 0.9	14.5 ± 1	17.6 ± 1.1	16.7 ±.8^∧^	18.2 ± 3.3
Age commence collision sport^*^	N/A	N/A	9.24 ±.8	17.6 ± 1.3	8.1 ± 0.5	17.0 ± 3.8
Years of collision sport participation^*^^#^	N/A	N/A	13.2 ± 0.8	5.6 ± 1.3	15.5 ± 0.8	9.0 ± 3.4
Number of previous concussions	N/A	N/A	N/A	N/A	2.9 ± 0.4	1.9 ± 0.4
Time since last concussion (years)	N/A	N/A	N/A	N/A	2.7 ± 0.4	5.1 ± 1.5

*Results are presented as Mean ± SEM*.

* main effect for Sex;

# main effect for history of concussion;

∧ *HoC males less than NoHoC females; p < 0.05. Results are presented as Mean ± SEM*.

### Serum Biomarker Abnormalities in Male Collision Sport Athletes

**Figure 1:**
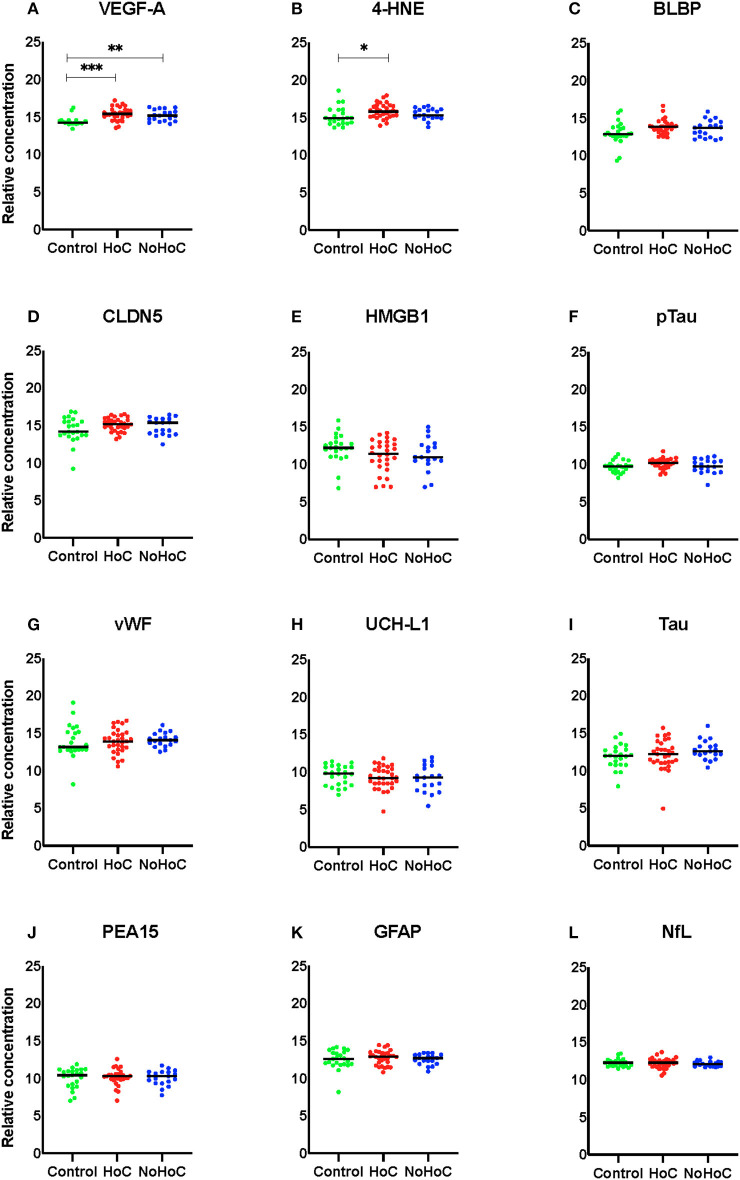
Serum protein levels in male participants. **(A)** Both the HoC and NoHoC groups had significantly greater concentrations of VEGF-A compared to the control group (**P < 0.01, ***P < 0.0001). **(B)** The HoC group had significantly greater concentrations of 4-HNE compared to the control group (*P < 0.05). **(C–L)** There were no other significant findings on the remaining markers.

Amongst males, Kruskal–Wallis tests identified significant between group differences for VEGF-A (*F* = 17.66, *p* = 0.001, *d* = 0.24; Figure 1A) and 4-HNE (*F* = 7.904, *p* = 0.019, *d* = 0.08; Figure 1B). For VEGF-A, *post-hoc* analysis found that both the HoC and NoHoC groups had significantly greater levels compared to the control group (*p* = 0.006; Figure 1A). For 4-HNE, the HoC group had significantly greater levels compared to the control group (*p* = 0.015). There was no other significant RPPM findings amongst the male group (effect sizes < 0.05; Figures 1C–L).

**Figure 2:**
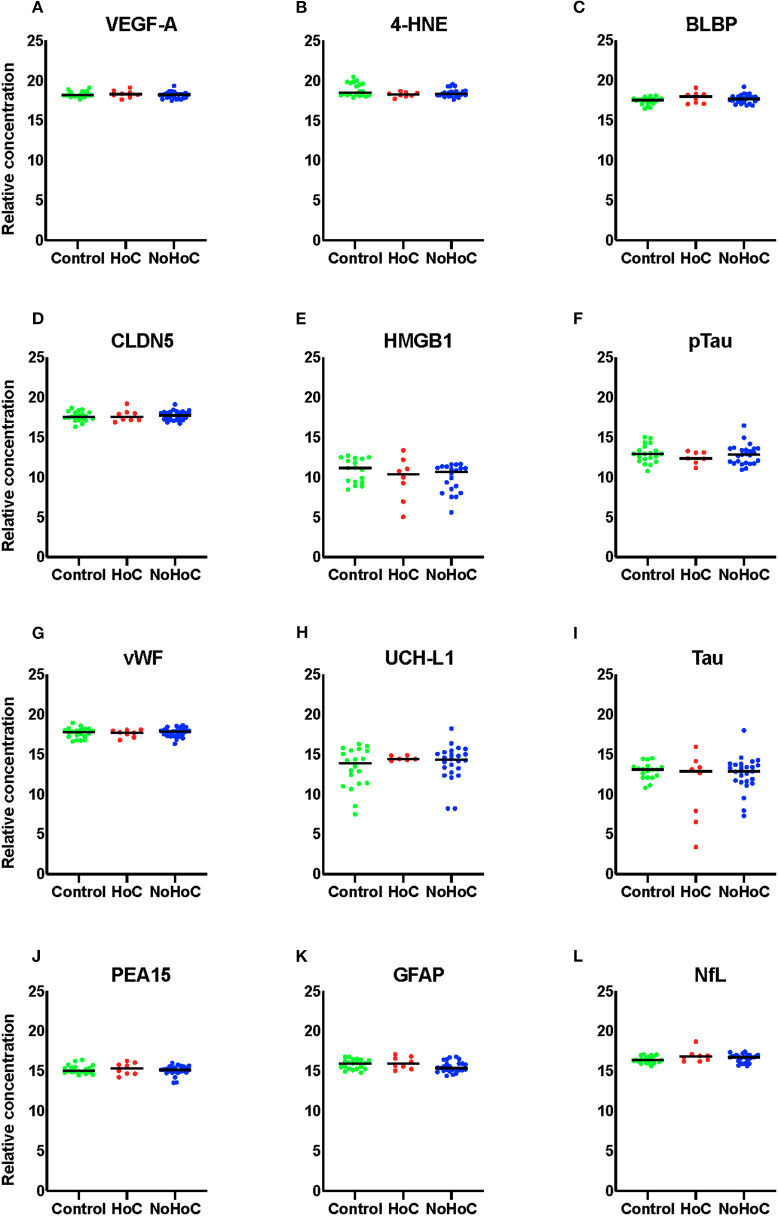
Serum protein levels in female samples of **(A)** VEGF-A, **(B)** 4-HNE, **(C)** BLBP, **(D)** CLDN5, **(E)** HMGB1, **(F)** pTau, **(G)** vWF, **(H)** UCH-L1, **(I)** Tau, **(J)** PEA-15, **(K)** GFAP, and **(L)** NfL. There were no significant findings on any of the markers.

As shown in Figure 2, there were no significant RPPM findings amongst the female groups (effect sizes <0.05).

### Relationships Between Serum Biomarker Levels and Collision Sport Exposure

The correlations matrix conducted to examine the relationships between biomarkers in male (Supplementary Table 1) and female (Supplementary Table 2) samples found multiple significant correlations between individual biomarkers.

Additionally, partial Spearman's correlation coefficients between years of collision sport and individual serum biomarker variables whilst controlling for biological age were completed (Supplementary Table 3). Partial Spearman's correlation coefficients between age of first exposure and individual serum biomarker variables whilst controlling for years of collision sport and biological age were also performed (Supplementary Table 3). For females, there was a correlation between fibrinogen levels and years of collision sport (0.521, *p* = 0.04). No other significant correlations were found.

## Discussion

This study investigated serum levels of protein biomarkers indicative of neuronal, astroglia, axonal and vascular injury, oxidative stress, and inflammation in male and female Australian rules footballers both with and without a HoC, as well as a control group of non-collision sport athletes with no history of neurotrauma. Male collision sport athletes, both with and without a HoC, had elevated levels of serum VEGF-A compared to non-collision sport playing controls. Male collision sport athletes with a HoC also had elevated levels of 4-HNE compared to non-collision sport playing controls. Levels of VEGF-A and 4-HNE did not correlate with measures of collision sport exposure (i.e., age commencing collision sport and years of collision sport participation). No significant between-group differences were reported for serum protein levels within female athletes, which may be related to the females having shorter collision sport careers compared to the males in this study.

VEGF-A has diverse biological functions including regulation of angiogenesis (i.e., the formation of new blood vessels), and elevated serum VEGF-A levels are indicative of endothelial stress and injury (17). Although some studies have reported increased VEGF-A levels in the blood following TBI, including mTBI (14, 15, 18–20), to the best of our knowledge, no studies have investigated the potential associations with collision sport participation or HoC. We found that VEGF-A concentration was increased in male, but not female, footballers when compared to their relative control athletes, but that this increase did not appear to be related to HoC. As such, although other factors are potentially involved, it is possible that a greater exposure to cumulative sub-concussive impacts in male footballers may have been a contributing factor. Supporting this hypothesis are the results of a recent neuroimaging study of male collegiate American football players, with Slobounov et al. (21) finding pre- and post-season differences in susceptibility weighted imaging (i.e., a measure of cerebrovascular integrity) in players exposed to RHIs, even in the absence of clinical symptoms or diagnosis of a concussion. We found no difference in serum levels of CLDN5, an endothelial tight junction protein between the control group and either groups of the footballers. Altered CLDN5 levels have been shown in various forms of traumatic brain injury (TBI) including repeated mTBI and thought to be associated with endothelial damage (22–24). Similar to CLDN5, serum levels of vWF were not different between the various groups of athletes further suggesting that male footballers more likely experience endothelial stress than damage, as elevated serum levels of vWF is associated with endothelial injury and microvascular bleeding after TBI. As VEGF-A is not brain-specific, differences in musculoskeletal trauma (25) and exercise intensity and frequency between collision and non-collision sports/sexes may have contributed to this finding (26). As such, further research is required to understand the mechanisms and potential significance of the VEGF-A increases observed in male footballers, and why this difference was not observed in females.

4-HNE is produced by oxidative stress-induced lipid peroxidation (27), and has shown to be elevated in circulation in neurodegenerative diseases including Parkinson's (28), Alzheimer's and amyotrophic lateral sclerosis (29), as well as following ischemic stroke (30). To the best of our knowledge no clinical studies have investigated circulating 4-HNE in the context of mTBI, however oxidative stress is thought to be prominent in this condition (29–31). Studies have found elevated circulating 4-HNE levels at 1 month following mTBI in rats (14, 15, 18). In the current study we found that serum levels of 4-HNE were significantly elevated in male footballers with a HoC when compared to controls, with no such differences observed in males with NoHoC and female footballers. As all measures were conducted in footballers without a concussion in the preceding 6 months, this finding may reflect a prolonged increase in oxidative stress due to concussion and sub concussive exposure. Importantly, as oxidative stress is not limited to the brain, systemic changes may have contributed to the elevated 4-HNE levels found in this population. Furthermore, although we detected no elevation in 4-HNE levels in footballers without a HoC, the lack of a control group with a HoC and no history of collision sport participation makes it difficult to conclude that collision sport participation was not a contributing factor.

There were no differences among the different groups of athletes in the serum levels of astroglial damage markers (GFAP, BLBP, PEA15), neuronal and axonal injury makers (UCH-L1, NfL, tau and p-tau) or HMGB1 (a marker of cellular damage and initiator of inflammatory response). These findings are congruent with previous findings reporting no association between a HoC and fluid biomarkers outside the acute (i.e., < 14 days) post-injury phase (32–34). For example, in a study of 415 athletes, there were no significant relationships between the number of previous concussions or cumulative head injury with baseline levels of serum biomarkers GFAP, S100B, and UCH-L1 (32). Alternatively, our findings may suggest that the RHIs in this particular group of collision sport athletes did not result in persisting or progressive damage to be detected in our current assay system, although changes may have been present if studied at a more acute timepoint after concussion or exposure to RHI, when pathophysiological disturbances may be more substantial. Moreover, with increasing evidence that RHI exposure may increase risk of chronic neurological disturbances, future studies are required to determine if the biomarkers assessed in this study may be altered at a more chronic stage of injury.

While we found evidence that male Australian rules footballers had elevated levels of VEGF-A and 4-HNE compared to non-collision athlete controls, there were no significant differences on any of the markers examined within the female groups. The interpretations regarding the serum levels between the sexes are limited as male and female samples were analyzed independent of each other and under slightly different conditions. With that said, there are a number of possible explanations for the different findings between males and females. There was a significant difference between male and female Australian rules footballers in terms of their exposure to collision sports. Specifically, males played more years of collision sport, and started at an earlier age, than females. Therefore, the increased levels of VEGF-A and 4-HNE might be attributed to the presumptive increased exposure to RHIs experienced by the males in this study. In support of this notion, a previous study found that cumulative RHI exposure in amateur American football players was predictive of depression and cognitive abnormalities later in life (32). However, other studies have failed to find a relationship between RHI exposure and neurological outcomes in American footballers (33), suggesting other factors may have contributed to our findings. For example, there are a number of preclinical studies (34), and initial human studies (6, 35, 36) indicating that males and females have inherent biological differences in their response to mTBI. A possible contributor to differences in circulating biomarkers between males and females are sex hormones, as well as fluctuation in hormones across the female menstrual cycle (37, 38). Furthermore, although we did not find differences within the female groups this may be due to the limited number of biomarkers used in this study, and other markers may have detected changes related to RHI exposure in females. Last, although we were unable to make direct comparisons between the sexes in this study, there appeared to be increased protein levels on many of the markers in females. This may be due to a number of factors including inherent biological differences or methodological reasons (39, 40). Future studies are therefore required to determine whether there are true basal differences on these markers between males and females, which would have important implications for clinical application. Furthermore, it would be interesting to explore how body mass might influence circulating blood protein levels, and whether this contributes to sex differences.

There are other limitations that should be considered when interpreting these findings. As alluded to above, some of the biomarkers examined in this study, including VEGF-A and 4-HNE, are not specific to the brain and could therefore reflect other systemic changes. Future studies are therefore required to determine the true origin of these abnormalities. Advances are being made toward developing brain-specific extracellular vesicle-derived blood biomarkers for brain injury (41, 42). Animal models that can control for central vs. peripheral injury could also be useful in this context (37, 43). Animal model studies controlling for brain injury severity could also help distinguish changes as a result of concussive vs. sub-concussive impacts. The clinical significance of elevated circulating VEGF-A and 4-HNE should also be considered. Future studies that incorporate detailed cognitive and neuropsychiatric measures, as well as longitudinal studies investigating how these changes predict long-term outcomes, would provide important clinical insights. Another limitation relates to the self-reported measures of HoC and history of sports participation, which have inherent issues with accuracy/bias. In addition, the number of females reporting a HoC was relatively low when compared to males, therefore further studies may be required to determine the impact of HoC on biomarker levels in females. A history of collision sports is also a surrogate measure of sub-concussive exposure, and future studies would benefit from using methods that can objectively measure impact exposure and force. It would have also been beneficial if we had recorded details related to time since most recent exercise/sport participation, as this may have influenced circulating non-brain specific biomarkers. Along these lines, more comprehensive details related to clinical and medical characteristics, as well as race and ethnicity, would have strengthened this study and allowed for further investigation into how these factors relate to biomarker outcomes. Finally, our use of a relatively large biomarker panel may result in false positives, and our findings should be replicated in larger cohorts.

In conclusion, this study found that male, but not female, Australian rules footballers had increased serum levels of VEGF-A and 4-HNE compared to non-collision athlete controls. The VEGF-A increase occurred independent of a HoC, while 4-HNE was only elevated in those with a HoC; however, neither VEGF-A and 4-HNE levels were found to correlate with measures of collision sport exposure. Although this study is not without its limitations, and further research is clearly required, our findings suggest that participation in collision sports may have persisting neurobiological consequences; that these consequences may differ between males and females; and that serum levels of VEGF-A and 4-HNE may be objective markers of these changes.

## Data Availability Statement

The raw data supporting the conclusions of this article can be made available by the authors upon request.

## Ethics Statement

The studies involving human participants were reviewed and approved by The Melbourne Health Human Ethics committee. The patients/participants provided their written informed consent to participate in this study.

## Author Contributions

BM, SM, DC, RM, TO'B, DA, and SS conceptualized and designed the study. BM, SM, WO'B, GS, MS, RB, JM, RA, I-HL, and ML were involved in participant recruitment and data collection. BM, SM, JM, RA, I-HL, DA, and SS were involved in data analysis. All authors contributed to the interpretation of the findings and writing of the manuscript.

## Conflict of Interest

The authors declare that the research was conducted in the absence of any commercial or financial relationships that could be construed as a potential conflict of interest.
