# Supplementary material for: Serum Protein Biomarker Findings Reflective of Oxidative Stress and Vascular Abnormalities in Male, but Not Female, Collision Sport Athletes
Source: Front Neurol. 2020 Sep 30;11:549624. doi: 10.3389/fneur.2020.549624 (PMC7561422; doi:10.3389/fneur.2020.549624)
Supplement: Supplementary file 1 [file Table_1.docx]

| **Supplementary Table 1.** ﻿Spearman Correlation Coefficients between serum protein levels of all biomarkers in male sample. *P < 0.05, **P < 0.01. |
| --- |
| \|  \| HNE4 \| BLBP \| CLDN5 \| Fibrinogen \| GFAP \| HMGB1 \| NFL \| PEA15 \| pTau \| Tau \| UCHL1 \| VEGFa \| \| --- \| --- \| --- \| --- \| --- \| --- \| --- \| --- \| --- \| --- \| --- \| --- \| --- \| \| BLBP \| .67^**^ \| 1 \| .743^**^ \| -.291^*^ \| 0.156 \| 0.038 \| .269^*^ \| .394^**^ \| .581^**^ \| 0.156 \| 0.116 \|  \| \| CLDN5 \| .81^**^ \| .74^**^ \| 1 \| -0.226 \| .270^*^ \| 0.198 \| .375^**^ \| .586^**^ \| .665^**^ \| .368^**^ \| 0.191 \|  \| \| Fibrinogen \| -.07 \| -.29^*^ \| -.22 \| 1 \| 0.175 \| -0.011 \| -0.167 \| -0.074 \| 0.018 \| .355^**^ \| -0.099 \|  \| \| GFAP \| .25^*^ \| .16 \| .27^*^ \| .17 \| 1 \| 0.235 \| 0.201 \| .269^*^ \| .291^*^ \| .333^**^ \| .310^**^ \|  \| \| HMGB1 \| .17 \| .04 \| .20 \| -.01 \| .23 \| 1 \| .266^*^ \| .295^*^ \| 0.168 \| .314^**^ \| 0.035 \|  \| \| NFL \| .51^**^ \| .27^*^ \| .37^**^ \| -.17 \| .20 \| .27^*^ \| 1 \| .368^**^ \| .333^**^ \| 0.188 \| 0.009 \|  \| \| PEA15 \| .48^**^ \| .39^**^ \| .59^**^ \| -.07 \| .27^*^ \| .29^*^ \| .37^**^ \| 1 \| .271^*^ \| 0.118 \| 0.152 \|  \| \| pTau \| .67^**^ \| .58^**^ \| .66^**^ \| .02 \| .29^*^ \| .17 \| .33^**^ \| .27^*^ \| 1 \| .337^**^ \| -0.004 \|  \| \| Tau \| .51^**^ \| .16 \| .37^**^ \| .35^**^ \| .33^**^ \| .31^**^ \| .19 \| .12 \| .34^**^ \| 1 \| 0.049 \|  \| \| UCHL1 \| -.03 \| .12 \| .19 \| -.10 \| .31^**^ \| .03 \| .01 \| .15 \| -.01 \| .05 \| 1 \|  \| \| VEGFa \| .82^**^ \| .68^**^ \| .79^**^ \| -.21 \| .30^*^ \| .04 \| .49^**^ \| .39^**^ \| .67^**^ \| .29^*^ \| .13 \|  \| \| vWF \| .71^**^ \| .45^**^ \| .63^**^ \| -.11 \| .38^**^ \| .36^**^ \| .51^**^ \| .41^**^ \| .42^**^ \| .60^**^ \| -.03 \| .58^**^ \| |
