# Supplementary material for: Serum Protein Biomarker Findings Reflective of Oxidative Stress and Vascular Abnormalities in Male, but Not Female, Collision Sport Athletes
Source: Front Neurol. 2020 Sep 30;11:549624. doi: 10.3389/fneur.2020.549624 (PMC7561422; doi:10.3389/fneur.2020.549624)
Supplement: Supplementary file 2 [file Table_2.docx]

| **Supplementary Table 2.** ﻿Spearman Correlation Coefficients between serum protein levels of all biomarkers in female sample. *P < 0.05, **P < 0.01. |
| --- |
| \|  \| HNE4 \| BLBP \| CLDN5 \| Fibrinogen \| GFAP \| HMGB1 \| NFL \| PEA15 \| pTau \| Tau \| UCHL1 \| VEGFa \| \| --- \| --- \| --- \| --- \| --- \| --- \| --- \| --- \| --- \| --- \| --- \| --- \| --- \| \| BLBP \| -.02 \|  \|  \|  \|  \|  \|  \|  \|  \|  \|  \|  \| \| CLDN5 \| .53** \| .28* \|  \|  \|  \|  \|  \|  \|  \|  \|  \|  \| \| Fibrinogen \| .31* \| .0005 \| .63** \|  \|  \|  \|  \|  \|  \|  \|  \|  \| \| GFAP \| .42** \| -0.04 \| .64** \| .42* \|  \|  \|  \|  \|  \|  \|  \|  \| \| HMGB1 \| .61** \| .18 \| .71** \| .41** \| .56** \|  \|  \|  \|  \|  \|  \|  \| \| NFL \| .15 \| 0.04 \| .44** \| .40** \| .45** \| .14 \|  \|  \|  \|  \|  \|  \| \| PEA15 \| .27 \| .46** \| .24 \| .16 \| .24 \| .33 \| .07 \|  \|  \|  \|  \|  \| \| pTau \| .69** \| 0.20 \| .82** \| .54** \| 0.72** \| .73 \| 0.43** \| .40** \|  \|  \|  \|  \| \| Tau \| .25 \| .04 \| .76** \| .61** \| .61** \| .42** \| .46** \| .12 \| .68** \|  \|  \|  \| \| UCHL1 \| -.21 \| .09 \| .38** \| .41** \| .41** \| .12 \| .38** \| .40** \| .38** \| .44** \|  \|  \| \| VEGFa \| .23 \| .26 \| .57** \| .16 \| .56** \| .54** \| .07 \| .46** \| .55** \| .37** \| .52** \|  \| \| vWF \| -.09 \| -.11 \| 0.24 \| .41** \| .25 \| .18 \| .26 \| .01 \| .13 \| .04 \| .15 \| -.02 \| |
