# Supplementary material for: Serum Protein Biomarker Findings Reflective of Oxidative Stress and Vascular Abnormalities in Male, but Not Female, Collision Sport Athletes
Source: Front Neurol. 2020 Sep 30;11:549624. doi: 10.3389/fneur.2020.549624 (PMC7561422; doi:10.3389/fneur.2020.549624)
Supplement: Supplementary file 3 [file Table_3.docx]

| **Supplementary Table 3. ﻿**Partial Spearman Correlation between serum protein levels of all biomarkers and participants athletic/concussion history in male and female sample. *P <.05. |
| --- |
| \|  \| Male \| \| \| \| \| \| \| \| \| \| \| \| \| \| --- \| --- \| --- \| --- \| --- \| --- \| --- \| --- \| --- \| --- \| --- \| --- \| --- \| --- \| \| HNE4 \| BLBP \| CLDN5 \| Fibrinogen \| GFAP \| HMGB1 \| NFL \| PEA15 \| pTau \| Tau \| UCHL1 \| VEGFa \| vWF \| \| Years of exposure \| .036 \| .147 \| -.059 \| .093 \| -.232 \| .103 \| -.197 \| -.037 \| .003 \| .169 \| -.276 \| -.054 \| .045 \| \| Age commence collision sport \| .014 \| -.019 \| -.025 \| -.067 \| .08 \| -.174 \| -.041 \| .014 \| .014 \| -.301 \| -.012 \| -.075 \| -.182 \| \|  \| Female \| \| \| \| \| \| \| \| \| \| \| \| \| \| HNE4 \| BLBP \| CLDN5 \| Fibrinogen \| GFAP \| HMGB1 \| NFL \| PEA15 \| pTau \| Tau \| UCHL1 \| VEGFa \| vWF \| \| Years of exposure \| -.293 \| .104 \| -.037 \| .087 \| -.145 \| -.090 \| -.209 \| -.145 \| -.172 \| .136 \| .048 \| -.156 \| .136 \| \| Age commence collision sport \| .158 \| -.33 \| .300 \| .521* \| .225 \| .136 \| .207 \| -.317 \| .327 \| .325 \| -.126 \| -.414 \| -.044 \| |
